# The Impact of Peer Stress on Disordered Eating Behaviors in Youths With Type 1 Diabetes: A Parallel Mediation Effect of Dual‐Mode Self‐Control

**DOI:** 10.1155/jdr/9964884

**Published:** 2026-04-27

**Authors:** Meijing Zhou, Yueting Qin, Hong Wang, Jian Yu, Min Zhu, Hong yun Zhu, Yang Lei, Jingjing Xu

**Affiliations:** ^1^ Department of Endocrinology, The First Affiliated Hospital with Nanjing Medical University, 300 Guangzhou Road, Nanjing, 210029, China, njmu.edu.cn; ^2^ School of Medical Imaging, Nanjing Medical University, 101 Longmian Avenue, Nanjing, 211166, China, njmu.edu.cn; ^3^ School of Nursing, Nanjing Medical University, 101 Longmian Avenue, Nanjing, 211166, China, njmu.edu.cn; ^4^ Department of Nursing, The First Affiliated Hospital with Nanjing Medical University, 300 Guangzhou Road, Nanjing, 210029, China, njmu.edu.cn; ^5^ Department of Epidemiology, Center for Global Health, School of Public Health, Nanjing Medical University, Nanjing, China, njmu.edu.cn

**Keywords:** disordered eating behaviors, dual-mode self-control, peer stress, type 1 diabetes

## Abstract

**Background:**

Disordered eating behaviors (DEBs) have been a prevalent issue among youths with type 1 diabetes (T1D), leading to impaired glycemic control, significant short‐ and long‐term complications, and elevated mortality risk. Peer stress plays a critical role in the development of DEBs in adolescents. However, the association between peer stress and DEBs in youths with T1D remains unestablished, and the underlying mechanisms are unclear. This study investigated how peer stress influences DEBs and examined the parallel mediating role of dual‐mode self‐control—comprising both impulsive and control systems—in this relationship.

**Methods:**

This cross‐sectional study included 180 youths with T1D who were recruited from two tertiary hospitals in Nanjing, China, between December 2021 and September 2022. Data were collected using the peer stress subscale of the Diabetes Stress Questionnaire for Youths, the Dual‐Mode Self‐Control Scale, and the Diabetes Eating Problem Survey‐Revised. Descriptive analyses and Pearson correlations were conducted in SPSS 22.0. Parallel mediation effects were tested via Model 4 of the PROCESS macro, using bias‐corrected bootstrap with 5000 resamples to determine significant mediation.

**Results:**

Peer stress significantly predicted DEBs (*B* = 0.55, *p* < 0.001). Only the impulsive system demonstrated partial mediation in the pathway from peer stress to DEBs (indirect effect = 0.13, 95% CI [0.04, 0.25]), accounting for 22.41% of the total effect.

**Conclusions:**

Peer stress directly influences DEBs and indirectly exacerbates DEBs through the activation of the impulsive system. These findings advance theoretical frameworks on peer stress and DEBs, informing interventions targeting peer stress reduction and impulse regulation to mitigate DEBs in youths with T1D.

## 1. Background

Type 1 diabetes (T1D) is a chronic autoimmune disorder characterized by the progressive destruction of pancreatic β‐cells, resulting in absolute insulin deficiency and a lifelong dependence on exogenous insulin therapy [1]. As T1D is predominantly diagnosed during childhood and adolescence, affected youths must manage complex daily self‐care tasks during a critical developmental period. These tasks include insulin administration, carbohydrate regulation, physical activity adjustment, and continuous glycemic monitoring. These intensive self‐management demands place adolescents with T1D at a heightened risk for disordered eating behaviors (DEBs), which refer to maladaptive eating‐ and weight‐related behaviors that compromise physical or psychological health [2–4]. Notably, insulin omission represents a T1D‐specific form of DEBs, allowing weight control without overt food restriction [5]. Our previous study identified a significant proportion (54.6%) of Chinese youths with T1D to be at high risk for DEBs [6]. Clinically, the co‐occurrence of T1D and DEBs is associated with poor glycemic control, an increased risk of diabetic ketoacidosis, and elevated psychiatric comorbidity [7, 8]. Given these profound health implications, identifying modifiable risk factors for DEBs remains an urgent priority in diabetes research and clinical care.

Peer stress has been recognized as a salient interpersonal stressor during adolescence, a developmental stage marked by heightened sensitivity to peer evaluation and social comparison [9]. For youths with T1D, peer stress may be particularly pronounced due to disease‐related visibility and stigma. Routine diabetes management behaviors—such as blood glucose monitoring and insulin injections in public settings—may attract unwanted attention or negative peer reactions, thereby intensifying feelings of social scrutiny and exclusion [10–12]. In addition, peer‐based body comparisons may exacerbate body dissatisfaction among youths with T1D, further increasing vulnerability to DEBs [13]. While research in nondiabetic populations has demonstrated a positive association between peer stress and DEBs [14–17], whether and how peer stress contributes to DEBs among youths with T1D remains insufficiently understood. Importantly, the unique behavioral options available to this population—particularly insulin omission—may alter both the manifestation of DEBs and their underlying psychological mechanisms.

Self‐control is a central regulatory capacity that enables individuals to inhibit impulsive responses and pursue long‐term goals, and it plays a critical role in health‐related behaviors requiring sustained regulation [18]. According to the dual‐system model, self‐control reflects the dynamic balance between an impulsive system that drives immediate reward seeking and a control system that supports deliberative regulation [19, 20]. Disruptions in this balance have been linked to various risk behaviors, including DEBs [21–24]. Stress exposure has been shown to activate impulsive tendencies while simultaneously impairing control capacity, thereby increasing vulnerability to maladaptive behaviors [25, 26]. In youths with T1D, this process may be amplified by the chronic self‐regulatory burden imposed by daily disease management, which can tax control resources over time. Under conditions of heightened peer stress, adolescents with T1D may therefore be especially prone to engaging in DEBs through concurrent activation of impulsive motivations (e.g., weight control, peer acceptance) and weakening of regulatory control. However, prior research has largely treated self‐control as a unidimensional construct, failing to capture the potentially divergent roles of impulsive and control systems in the stress–DEB pathway [27].

This study aims to examine the association between peer stress and DEBs among youths with T1D, with a specific focus on the mediating role of dual‐mode self‐control. By situating peer stress within the unique social context of T1D and conceptualizing self‐control as an interaction between impulsive and control systems, this study offers a nuanced understanding of the psychological mechanisms underlying DEBs in adolescents with T1D and extends existing research beyond nondiabetic populations.

## 2. Methods

### 2.1. Participants

**Figure 1:**
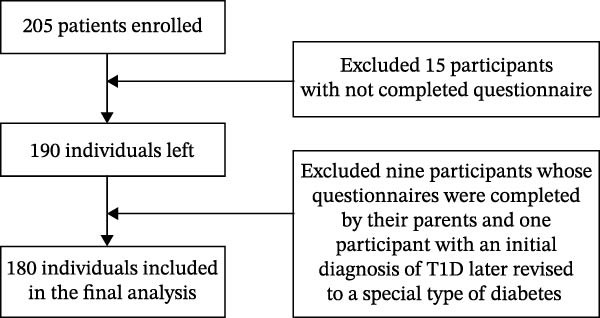
Flowchart of patient inclusion.

Between December 2021 and September 2022, youths with T1D were recruited via convenience sampling from two public tertiary hospitals in Nanjing, China. The inclusion criteria were: (1) a confirmed T1D diagnosis for more than 6 months; (2) age between 10 and 24 years; and (3) the ability to understand and complete the survey in Chinese. Based on their medical records, individuals with a diagnosed eating disorder, other chronic illnesses (e.g., arthritis, malignancies), or co‐occurring psychiatric conditions were excluded. Notably, participants who scored 20 or above on the Diabetes Eating Problem Survey‐Revised (DEPS‐R) were referred to a psychiatrist for further evaluation. Those diagnosed with a clinical eating disorder following assessment were excluded from the final sample. The required sample size was determined using 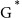
Power 3.1 software, applying linear multiple regression (*F*‐tests) as the statistical model. Parameters included a two‐tailed significance level (*α*) of 0.05, an anticipated effect size of 0.10, and a statistical power of 0.80. Eleven predictors were specified, including diabetes distress, dual‐mode self‐control, demographic variables, and disease‐related characteristics. Based on these inputs, the minimum sample size required was calculated to be 179. Of the 205 eligible patients enrolled, 25 were excluded due to incomplete questionnaires (*n* = 15), questionnaires completed by parents (*n* = 9), and an initial diagnosis of T1D later revised to a special type of diabetes (*n* = 1), resulting in a final analytical sample of 180 participants. A flowchart summarizing participant recruitment, exclusion, and final inclusion is presented in Figure 1.

### 2.2. Data Collection

To ensure that every participant could understand the questionnaire and complete it independently, we conducted a pre‐survey. Structured paper‐and‐pencil questionnaires were administered to the participants. Any inquiries participants had about the questionnaires were to be addressed by the investigators. Each questionnaire took approximately 20 min, after which they were collected and examined promptly. The investigators chose five items at random to determine the comprehension of participants between 10 and 13 years of age. To ensure data quality, investigators and the participants verified each item in the questionnaires individually in case any misunderstandings arose. Importantly, participants were not informed beforehand that their questionnaires would be examined. Each participant used an electronic scale and a stadiometer to measure their height and body weight after finishing the questionnaire. The body mass in kilograms divided by the square of the height in meters yields the body mass index (BMI).

### 2.3. Ethics Approval

This study received ethical approval from the Ethics Committee of the First Affiliated Hospital with Nanjing Medical University (Approval Number 2021‐NT49). All procedures adhered to the Declaration of Helsinki to safeguard participants’ rights, privacy, and welfare. Informed consent was obtained from each participant following full disclosure of the study’s purpose, procedures, potential risks and benefits, and voluntary nature, ensuring their unconditional right to withdraw at any time without affecting their therapy. Parental consent was additionally secured for participants under 18 years of age. To ensure confidentiality, all personally identifiable information was removed during the initial data processing, and the collected data were anonymized using unique coded identifiers. These anonymized datasets were securely stored with access restricted by password to the research team. These protocols rigorously followed international ethical standards for human subjects research, specifically protecting participant autonomy through informed consent and data security.

### 2.4. Instruments

The questionnaire included demographic information, the Diabetes Stress Questionnaire for Youths (DSQY), the Dual Mode Self‐Control Scale (DMSC‐S), and the DEPS‐R. Disease information was acquired from medical records.

### 2.5. The Diabetes Stress Questionnaire for Youths

We used the peer stress subscale of the DSQY, developed by Delamater AM et al. [9], to assess peer‐related diabetes stress perceived by youths with T1D. This subscale comprises eight items rated on a four‐point Likert scale (0 = no pressure, 3 = severe pressure). The total score is derived by summing the individual item scores, with higher scores indicating greater perceived peer stress. The peer stress subscale of the DSQY has demonstrated good psychometric properties in Chinese populations with T1D, with a Cronbach’s *α* of 0.92 [28]. In this study, its Cronbach’s *α* was 0.955.

### 2.6. The Dual Mode Self‐Control Scale

Dual‐mode self‐control was evaluated using the DMSC‐S. This scale was designed by Hofmann W et al. and translated by Xie D et al. [20, 29]. The DMSC‐S consists of two subscales: control (nine items) and impulse system (12 items). Each item employs a five‐point Likert scale, with responses ranging from “1 point” for total disagreement to “5 points” for complete agreement. Subscale scores were calculated by summing the corresponding item scores, with higher scores indicating a stronger control or impulse system. All items were scored in the same direction, and no reverse‐scored items were included. Previously validated in Chinese youths with excellent psychometric properties [29], confirmatory factor analysis confirmed its dual‐system structure with good fit indices: RMSEA = 0.06, NFI = 0.92, NNFI = 0.92, CFI = 0.93. Reliability analyses showed Cronbach’s *α* of 0.82 (full scale), 0.74 (control system), and 0.80 (impulse system). In this study, the Cronbach’s *α* for the DMSC‐S was 0.759, with values of 0.803 and 0.927 for the control and impulse systems, respectively.

### 2.7. The Disordered Eating Behaviors Scale‐Revised

The DEBS‐R was employed to assess DEBs among youths with T1D. This 16‐item scale is a revised version of a measure developed by Antisdel et al. [30]. Items are rated from never (0 points) to always (5 points). The total score is calculated by summing all item responses, with higher scores indicating more severe DEBs. Following the original validation studies, a total score >20 was recommended as the clinical cutoff to identify individuals exhibiting a higher frequency and greater severity of disordered eating symptoms and who may require further clinical assessment [7, 30]. No items on the DEPS‐R are reverse‐scored. Wencong Lv et al. [31] adapted this scale into Chinese and established its sound psychometric properties in youths with T1D in China, reporting a Cronbach’s *α* values of 0.85. In this study, the Cronbach’s α of DEBS‐R was 0.823.

### 2.8. Statistical Analysis

All statistical analyses were conducted using SPSS version 22.0. To ensure data quality and the validity of the results, stringent procedures were adopted for data processing and validation. The normality of continuous variables was assessed through skewness, kurtosis, and Q–Q plots. To identify potential common method bias that could compromise the reliability of the findings, Harman’s single‐factor test was performed. Multicollinearity among independent variables was examined using variance inflation factors (VIF). Descriptive statistics were reported as means and standard deviations for continuous variables, and as frequencies and percentages for categorical or ordinal variables. Independent *t*‐tests and one‐way analyses of variance (ANOVA) were conducted to examine differences in peer stress, DEBs, and dual‐mode self‐control across sociodemographic and disease‐related characteristics. Based on prior literature, relevant sociodemographic and disease‐related variables were identified a priori as potential covariates. These variables were then examined using univariate analyses to explore their associations with peer stress, dual‐mode self‐control, and DEBs. Subsequently, their effects on the proposed mediation pathway were evaluated using hierarchical regression analyses, and variables demonstrating significant effects in these models were retained as covariates in the final mediation analyses. Bivariate Pearson correlations among dual‐mode self‐control, DEBs, and peer stress were examined. Hierarchical multiple regression analyses were performed to test whether dual‐mode self‐control simultaneously mediated the relationship between peer stress and DEBs. The parallel mediation model was tested using Model 4 of the PROCESS macro in SPSS, which estimates total, direct, and indirect effects. Bootstrapping with 5000 resamples was used, and statistical significance was determined by 95% confidence intervals (CIs) that did not include zero.

## 3. Results

### 3.1. Assessment of Normality, Common Method Bias, and Variance Inflation Factors

Q–Q plots indicated that the continuous variables closely conformed to normal distribution patterns. The absolute values of kurtosis ranged from 0.144 to 1.141, while the absolute values of skewness ranged from 0.305 to 0.854. As noted by Drezner [32], strict adherence to normality is uncommon in real‐world data. However, data are generally considered approximately normal when the absolute value of kurtosis is below 10 and skewness is below 3 [33]. Based on these criteria, the present dataset meets the acceptable standards for normality. Additionally, all VIF values were well below the commonly accepted threshold of 5.0, indicating no serious multicollinearity concerns.

To assess potential common method bias, Harman’s single‐factor test was conducted. The analysis extracted 10 factors with eigenvalues greater than 1, and the first factor accounted for 25.16% of the total variance—well below the 40% threshold—suggesting that common method bias was not a significant issue in this study [34].

### 3.2. Descriptive Analyses

A total of 180 questionnaires were analyzed (Table 1). Participants had a mean age of 19.26 ± 3.94 years, with 56.67% being females. Among the participants, 14.45% were overweight and 13.33% were underweight. Education levels showed that 48.33% had completed higher education and 44.44% had completed secondary education. Additionally, 43.89% had lived with T1D for more than 5 years, while 53.33% used insulin pumps for glucose management. Regarding family income, 32.22% reported a monthly household income exceeding RMB 10,000. Significant differences in DEBs were observed by gender and place of residence. Female participants reported higher DEBs scores than males (24.51 ± 10.78 vs. 18.62 ± 9.32, *p* < 0.001). DEBs scores also differed significantly by place of residence, with higher scores observed among participants living in towns and rural areas compared with those residing in cities (city: 19.78 ± 9.77; town: 25.28 ± 10.81; countryside: 23.57 ± 11.05; *p* = 0.012). Similarly, significant differences in peer stress were observed across age groups and educational levels (*p* < 0.05), whereas no significant differences were detected in either the control system or the impulsive system according to sociodemographic or disease‐related characteristics.

**Table 1:** Sociodemographic and clinical characteristics of the participants, as well as the distributions of disordered eating behaviors in categorical items (*N* = 180).

Characteristics	*N* (%) of participants	Peer stress	Impulse system	Control system	Disordered eating behaviors
Sex					
Male	78 (43.33)	7.13 (6.11)	27.22 (8.38)	32.88 (4.30)	18.62 (9.32)
Female	102 (56.67)	7.54 (6.63)	28.24 (9.44)	31.77 (4.62)	24.51 (10.78)
*p*‐Value	—	0.670	0.449	0.102	<0.001
Age					
＜18	63 (35.00)	9.05 (6.40)	28.95 (8.29)	32.03 (4.75)	21.05 (10.59)
≥18	117 (65.00)	6.45 (6.23)	27.18 (9.32)	32.38 (4.39)	22.44 (10.55)
*p*‐Value	—	0.009	0.208	0.626	0.400
BMI classification					
Underweight	24 (13.33)	7.21 (6.83)	25.21 (8.15)	32.88 (5.80)	20.96 (15.05)
Normal	130 (72.22)	7.53 (6.28)	28.59 (9.11)	32.12 (4.30)	22.05 (9.48)
Overweight or obese	26 (14.45)	6.65 (6.76)	26.23 (8.81)	32.35 (4.36)	22.38 (11.16)
*p*‐Value	—	0.811	0.150	0.752	0.930
Education					
Primary education	13 (7.23)	7.85 (6.73)	29.15 (8.95)	31.46 (5.11)	20.69 (7.43)
Secondary education	80 (44.44)	8.81 (6.62)	29.10 (9.33)	31.91 (4.69)	22.94 (12.37)
Higher education	87 (48.33)	5.95 (5.88)	26.40 (8.56)	32.69 (4.25)	21.24 (9.06)
*p*‐Value	—	0.014	0.131	0.436	0.544
Residence					
City	93 (51.67)	6.97 (6.43)	26.45 (7.69)	33.03 (4.64)	19.78 (9.77)
Town	36 (20.00)	6.97 (5.54)	29.17 (9.57)	31.31 (4.04)	25.28 (10.81)
Countryside	51 (28.33)	8.35 (6.90)	29.29 (10.47)	31.51 (4.40)	23.57 (11.05)
*p*‐Value	—	0.427	0.114	0.056	0.012
Diabetes duration (year)					
0.5–1	24 (13.33)	6.54 (6.30)	28.00 (7.84)	31.58 (4.61)	20.50 (8.10)
1–3	41 (22.78)	9.00 (6.00)	28.07 (9.73)	31.56 (4.98)	20.66 (12.74)
3–5	36 (20.00)	7.61 (7.20)	29.50 (10.42)	31.89 (4.94)	22.17 (10.29)
>5	79 (43.89)	6.65 (6.18)	26.82 (8.23)	32.99 (3.97)	22.97 (10.16)
*p*‐Value	—	0.249	0.522	0.283	0.613
Family Monthly income (yuan)					
<3000	13(7.22)	11.00(8.32)	33.08 (9.40)	30.46 (4.24)	22.62 (9.51)
3000–5000	39(21.67)	6.77(6.56)	28.87 (10.25)	31.92 (4.42)	22.97 (10.36)
5000–10,000	70(38.89)	7.70(6.12)	27.33 (8.76)	32.14 (4.53)	22.40 (9.98)
>10,000	58 (32.22)	6.53 (5.98)	26.47 (7.94)	33.02 (4.56)	20.59 (11.66)
*p*‐Value	—	0.126	0.088	0.266	0.682
Insulin regimen					
Insulin pump	96 (53.33)	8.01 (6.85)	27.67 (8.90)	32.30 (4.23)	21.74 (10.76)
Insulin pen	84 (46.67)	6.62 (5.78)	27.95 (9.04)	32.20 (4.84)	22.20 (10.38)
*p*‐Value	—	0.146	0.832	0.883	0.770

*Note*: BMI categories were determined using age‐appropriate Chinese reference standards. For participants aged 18 years or older, BMI was classified as underweight (BMI < 18.5 kg/m^2^), normal weight (18.5 kg/m^2^ ≤ BMI < 24.0 kg/m^2^),overweight (24 kg/m^2^ ≤ BMI < 28 kg/m^2^), or obesity (BMI ≥ 28 kg/m^2^) [35]. For participants younger than 18 years, BMI categories (underweight, normal weight, or overweight/obesity) were defined based on sex‐ and age‐specific cutoffs in the Chinese screening criteria for malnutrition, overweight, and obesity among school‐aged children and adolescents [36, 37].

### 3.3. Pearson Correlation Among Peer Stress, Dual‐Mode Self‐Control and Disordered Eating Behaviors

Table 2 shows that DEBs were positively correlated with the impulse system (*r* = 0.443, *p* < 0.01) and peer stress (*r* = 0.338, *p* < 0.01), but negatively correlated with the control system (*r* = −0.372, *p* < 0.01). Furthermore, peer stress was positively associated with the impulse system (*r* = 0.273, *p* < 0.01) and negatively associated with the control system (*r* = −0.198, *p* < 0.01).

**Table 2:** Pearson correlations among peer stress, impulse system, control system, and disordered eating behaviors.

Variable	1	2	3	4
1. Peer stress	1	—	—	—
2. Impulse system	0.273 ^∗∗^	1	—	—
3. Control system	−0.198 ^∗∗^	−0.440 ^∗∗^	1	—
4. Disordered eating behaviors	0.338 ^∗∗^	0.443 ^∗∗^	−0.372 ^∗∗^	1

*Note*:  ^∗∗^ *p* < 0.01.

### 3.4. The Parallel Mediating Role of Dual‐Mode Self‐Control Between Peer Stress and Disordered Eating Behaviors

We employed hierarchical regression analysis to test the parallel mediation effect of dual‐mode self‐control on the relationship between peer stress and DEBs. Based on prior literature and preliminary univariate analyses, age, gender, education, and place of residence were identified as relevant covariates and were therefore included in the hierarchical regression analyses. As shown in Table 3, a series of hierarchical linear regression models was constructed to examine the proposed mediation framework.

**Table 3:** Hierarchical linear regression model for predicting the impulse system and control system and disordered eating behaviors.

	Impulse system				Control system				Disordered eating behaviors					
	Model 1		Model 2		Model 1		Model 2		Model 1		Model 2		Model 3	
Control variables	*B* (SE)	*p*	*B* (SE)	*p*	*B* (SE)	*p*	*B* (SE)	*p*	*B* (SE)	*p*	*B* (SE)	*p*	*B* (SE)	*p*
Sex														
Male	1.06 (1.36)	0.436	0.87 (1.32)	0.513	−1.08 (0.68)	0.115	−1.01 (0.67)	0.136	5.97 (1.52)	<0.001	5.66 (1.43)	<0.001	5.01 (1.32)	<0.001
Female	(Omitted)	—	(Omitted)	—	(Omitted)	—	(Omitted)	—	(Omitted)	—	(Omitted)	—	(Omitted)	—
Age														
<18	−0.04 (2.00)	0.984	0.50 (1.96)	0.799	−0.39 (1.00)	0.701	−0.58 (0.99)	0.558	3.75 (2.24)	0.095	4.62 (2.11)	0.030	4.25 (1.94)	0.003
≥18	(Omitted)	—	(Omitted)	—	(Omitted)	—	(Omitted)	—	(Omitted)	—	(Omitted)	—	(Omitted)	—
Education														
Primary education	3.24 (3.30)	0.329	3.07 (3.21)	0.341	−1.09 (1.65)	0.250	−1.84 (1.63)	0.260	3.89 (3.68)	0.292	3.62 (3.46)	0.297	1.94 (3.20)	0.545
Secondary education	2.83 (1.85)	0.128	2.10 (1.82)	0.250	−1.11 (0.93)	0.233	−0.84 (0.92)	0.364	4.46 (2.07)	0.033	3.27 (1.96)	0.097	2.27 (1.81)	0.212
Higher education	(Omitted)	—	(Omitted)	—	(Omitted)	—	(Omitted)	—	(Omitted)	—	(Omitted)	—	(Omitted)	—
Residence														
City	−2.99 (1.57)	0.059	−2.42 (1.54)	0.117	1.46 (0.79)	0.065	1.26 (0.78)	0.110	−2.89 (1.75)	0.101	−1.99 (1.66)	0.233	−0.73 (1.54)	0.638
Town	−0.48 (1.97)	0.808	0.15 (1.92)	0.939	−0.20 (0.98)	0.838	−0.43 (0.97)	0.659	1.94 (2.20)	0.378	2.95 (2.07)	0.157	2.75 (1.91)	0.151
Countryside	(Omitted)	—	(Omitted)	—	(Omitted)	—	(Omitted)	—	(Omitted)	—	(Omitted)	—	(Omitted)	—
Independent variable: Peer stress	—	—	0.34 (0.11)	0.001	—	—	−0.13 (0.05)	0.020	—	—	0.55 (0.11)	<0.001	0.39 (0.11)	<0.001
Mediator 1: Impulse system	—	—	—	—	—	—	—	—	—	—	—	—	0.34 (0.08)	<0.001
Mediator 2: Control system	—	—	—	—	—	—	—	—	—	—	—	—	−0.35 (0.16)	0.031
	*R* ^2^ = 0.052		*R* ^2^ = 0.107		*R* ^2^ = 0.058		*R* ^2^ = 0.087		*R* ^2^ = 0.144		*R* ^2^ = 0.248		*R* ^2^ = 0.372	
	Adjusted *R* ^2^ = 0.019		Adjusted *R* ^2^ = 0.071		Adjusted *R* ^2^ = 0.025		Adjusted *R* ^2^ = 0.050		Adjusted *R* ^2^ = 0.114		Adjusted *R* ^2^ = 0.217		Adjusted *R* ^2^ = 0.339	
	*F* (6, 173) = 1.583		*F* (1, 172) = 2.940		*F* (6, 173) = 1.765		*F* (1, 172) = 2.339		*F* (6, 173) = 4.846		*F* (1, 172) = 8.083		*F* (2, 170) = 11.190	
	*p* < 0.001		*p* < 0.001		*p* < 0.001		*p* < 0.001		*p* = 0.008		*p* < 0.001		*p* < 0.001	

In Model 1, age, sex, education level, and place of residence were entered as control variables in three separate regression models, with the impulse system, control system, and DEBs specified as the dependent variables, respectively. When DEBs were specified as the dependent variable, sex was the only factor that showed a statistically significant association with DEBs (*B* = 5.97, *p*  < 0.001). No significant associations were observed between the control variables and either the impulse system or the control system.

Subsequently, peer stress was added as the independent variable to each Model 1, with the impulse system, control system, and DEBs specified as the dependent variables, respectively (Model 2). Peer stress was positively associated with the impulse system (*B* = 0.34, *p* = 0.001) and negatively associated with the control system (*B* = −0.13, *p* = 0.020). No significant associations were observed between the control variables and either the impulse system or the control system.

**Figure 2:**
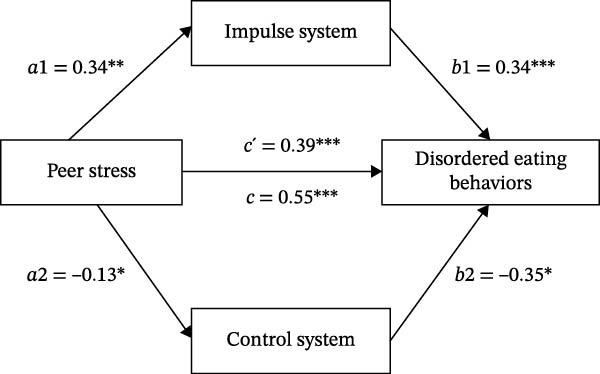
The paths of the parallel mediation model. Unstandardized regression coefficients are displayed. *a*1: the direct effect of peer stress on the impulse system; *a*2: the direct effect of peer stress on the control system; *b*1: the direct effect of the impulse system on DEBs; *b*2: the effect of the control system on DEBs; *c’*: the direct effect of peer stress on DEBs; *c*: the total effect of peer stress on DEBs.  ^∗^ *p* < 0.05;  ^∗∗^ *p* < 0.01;  ^∗∗∗^ *p* < 0.001.

In the model predicting DEBs, both sex (*B* = 5.66, *p*  < 0.001) and age (*B* = 4.62, *p* = 0.030) showed statistically significant effects, and peer stress remained a significant predictor of DEBs (*B* = 0.55, *p*  < 0.001). Model 3 further incorporated the impulse system and the control system into the regression model predicting DEBs. In this model, both sex (*B* = 5.01, *p*  < 0.001) and age (*B* = 4.25, *p* = 0.003) remained significantly associated with DEBs and were therefore retained as covariates in the final mediation model. The impulse system was positively associated with DEBs (*B* = 0.34, *p*  < 0.001), whereas the control system was negatively associated with DEBs (*B* = −0.35, *p* = 0.031). Meanwhile, the regression coefficient for peer stress was attenuated but remained statistically significant (*B* = 0.39, *p*  < 0.001), indicating that the impulse system and control system jointly and partially mediated the association between peer stress and DEBs. The paths of the parallel mediation model are presented in Figure 2.

Using SPSS 22.0 (PROCESS Macro Model 4), we verified parallel mediation effects (Table 4), with sex and age retained as covariates. Bias‐corrected bootstrap analysis demonstrated that peer stress exerted a significant indirect effect on DEBs through the impulse system, with an effect size of 0.13 accounting for 22.41% of the total effect (95% CI [0.04, 0.25]). In contrast, the control system’s indirect effect was nonsignificant (95% CI [−0.00, 0.14]).

**Table 4:** The bootstrap results of parallel mediation analysis.

Parallel mediation	Effect	SE	95% CI
Total effect (Peer stress → Disordered eating behaviors)	0.58	0.11	(0.36, 0.81)
Direct effect (Peer stress → Disordered eating behaviors)	0.40	0.11	(0.18, 0.61)
Indirect effect			
Peer stress → Impulse system → Disordered eating behaviors	0.13	0.06	(0.04, 0.25)
Peer stress → Control system → Disordered eating behaviors	0.05	0.04	(−0.00, 0.14)

## 4. Discussion

This study provides the first empirical evidence elucidating the mechanism by which peer stress influences DEBs. Our findings demonstrate that the association between peer stress and DEBs is exclusively mediated through the impulse system.

Our findings are consistent with prior research indicating that peer stress contributes to DEBs. For instance, Libbey et al. [16] demonstrated that frequent peer teasing significantly correlates with increased DEBs and reduced self‐esteem. Similarly, Beckers et al. [17] identified interpersonal peer problems—including victimization, rejection, and friendship deficits—as significant predictors of DEBs. These findings are especially relevant during adolescence and emerging adulthood, developmental stages marked by heightened sensitivity to peer evaluation, identity formation, and autonomy‐seeking [10]. Among youths, peer pressure often arises from the desire for peer acceptance, appearance‐ and behavior‐based social comparisons, and pressures to conform to group norms [38]. Critically, for youths with T1D, disease‐specific behaviors—such as insulin injections and glucose monitoring—can be visible and stigmatizing, triggering interpersonal challenges like social exclusion, embarrassment, and loneliness [39]. These social stressors can amplify the emotional burden of peer stress. According to problem‐behavior theory, adolescent behaviors result from the interaction between individual characteristics (e.g., values, beliefs) and environmental influences (e.g., peer stress) [40]. Within this framework, peer pressure may intensify internal conflict and self‐consciousness, thereby increasing susceptibility to maladaptive coping strategies such as DEBs. For youths with T1D, DEBs may not only serve as a means to control body weight and manage emotional distress but also function as a strategy for impression management—for example, avoiding diabetes‐related behaviors in public to minimize stigma and maintain peer acceptance. This finding underscores peer stress as a modifiable target for DEB interventions in youths with diabetes.

When examining the specific impact of peer stress on DEBs, our results indicated that only the impulsive system served as a significant mediator. This finding is consistent with a study by Hamilton et al. [41], which demonstrated that impulsivity mediated the relationship between stress and alcohol use. According to general strain theory, individuals who lack effective coping strategies tend to engage in impulsive behaviors to obtain immediate relief from emotional distress [42]. Among youths with T1D, DEBs may not only function as maladaptive coping strategies to alleviate emotional discomfort but also as attempts to conform to perceived social norms or regain a sense of control. Neurobiological evidence indicates that risk‐taking peaks during adolescence, driven by preferential activation of the early‐maturing impulsive system (socioemotional‐incentive processing network), which amplifies the appeal of novelty, excitement, and reward at a developmental stage when the still‐maturing cognitive control system lacks sufficient inhibitory capacity [43]. Moreover, chronic stress exposure, such as peer stress, exacerbates this imbalance by weakening prefrontal executive control and sensitizing limbic reward circuitry, thereby biasing decision‐making toward immediate gratification [44]. It is unsurprising, then, that only the impulsive system—but not the control system—mediated the relationship between peer stress and DEBs in our study. Future research should employ longitudinal and neurocognitive approaches to determine whether the control system might also serve as a mediator in the pathway from peer stress to DEBs. A deeper understanding of these mechanisms could guide targeted interventions that strengthen cognitive control and reduce reliance on impulsive coping strategies among youths with T1D.

### 4.1. Limitations and Practical Implications

Although this study is among the first to examine the association between peer stress and DEBs and to explore the underlying mechanisms in youths with T1D, several limitations should be acknowledged. First, although the cross‐sectional mediation analysis provides support for our hypothesis, it does not allow for causal inference. Future longitudinal studies are necessary to verify the proposed directional pathways Second, the sample was drawn from two medical centers in Nanjing, which may limit the generalizability of the findings. Replication in more diverse geographic and clinical settings is needed to enhance external validity. Third, although the self‐report instruments demonstrated satisfactory psychometric properties, they are subject to recall bias, social desirability effects, and other limitations inherent in self‐report methods. To improve data reliability, future research should adopt multi‐informant and multi‐method strategies, including clinical interviews, behavioral tasks, and reports from parents or teachers. Fourth, our study focused on peer stress, dual‐mode self‐control, and DEBs, without examining other relevant variables such as body dissatisfaction or social media use. Including these factors in future models may provide a more comprehensive understanding of the pathways to DEBs among youths with T1D.

## 5. Conclusion

This study investigated the parallel mediating role of dual‐mode self‐control in the relationship between peer stress and DEBs. The findings revealed that peer stress increased the risk of DEBs primarily through the impulsive system. These results underscore peer pressure as a modifiable risk factor and highlight the importance of supporting adolescents’ self‐control capacities and coping strategies. Future research should expand to more diverse and representative populations to determine whether the observed patterns generalize across different cultural or clinical settings. Additionally, future studies could explore additional psychological variables, such as body dissatisfaction and social media exposure, which might interact with peer stress to exacerbate DEBs. A deeper understanding of these psychosocial mechanisms will provide insights for developing more targeted and developmentally appropriate interventions aimed at improving mental and physical health outcomes in youths with T1D.

**Nomenclature**
DEBs: Disordered eating behaviors
T1D: Type 1 diabetes
DSQY: Diabetes stress questionnaire for youths
DMSC‐S: Dual mode self‐control scale
DEPS‐R: Diabetes eating problem survey‐revised
SD: Standard deviation
CI: Confidence interval.

## Author Contributions

**M.J.Z.:** Conceptualization, methodology, formal analysis, writing. **H.W., J.Y., H.Y.Z. and M.Z.:** investigation. **Y.T.Q.:** conceptualization and formal analysis. **Y.L.**: writing and project administration. **J.J.X.:** review and editing, supervision, project administration.

## Funding

This study was supported by the College Student Innovation and Entrepreneurship Training Program of Jiangsu Province (Grant 202410312065Z), Clinical ability improvement project from the First Affiliated Hospital with Nanjing Medical University (Grant JSPH‐NB‐2022‐12), China Social Welfare Foundation‐Nursing Innovation Support Project (Grant HLCXKT‐20230702), Chinese Medical Association Journals Nursing Research project (Grant CMAPH‐NRD2022038), Project of “Nursing Science” Funded by the 4th Priority Discipline Development Program of Jiangsu Higher Education Institutions (Jiangsu Education Department [2023], Grant 11), Innovation and Application of Chronic Disease Management and Treatment Technology, Project of the China Medical Foundation (Grant 2026CMFC8), 2023 Medical Research Project of the Health Commission of Jiangsu Province (Grant H2023110) and Sweet Doctor Cultivation Project of the Hunan Sinocare Diabetes Public Welfare Foundation (Grant 2023SD01).

## Ethics Statement

This study received ethical approval from the Ethics Committee of the First Affiliated Hospital with Nanjing Medical University (Approval Number 2021‐NT49). All procedures adhered to the Declaration of Helsinki to safeguard participants’ rights, privacy, and welfare. Informed consent was obtained from each participant following full disclosure of the study’s purpose, procedures, potential risks and benefits, and voluntary nature, ensuring their unconditional right to withdraw at any time without affecting their therapy. Parental consent was additionally secured for participants under 18 years of age. To ensure confidentiality, all personally identifiable information was removed during initial data processing, and the collected data were anonymized using unique coded identifiers. These anonymized datasets were stored securely with access restricted by password to the research team. These protocols rigorously followed international ethical standards for human subjects research, specifically protecting participant autonomy through informed consent and data security.

## Consent

The authors have nothing to report.

## Conflicts of Interest

The authors declare no conflicts of interest.

## Data Availability

The data from this study are available from the corresponding author upon reasonable request.
